# Can a penalized-likelihood estimation algorithm be used to reduce the injected dose or the acquisition time in ^68^Ga-DOTATATE PET/CT studies?

**DOI:** 10.1186/s40658-021-00359-6

**Published:** 2021-02-12

**Authors:** Alexandre Chicheportiche, Elinor Goshen, Jeremy Godefroy, Simona Grozinsky-Glasberg, Kira Oleinikov, Amichay Meirovitz, David J. Gross, Simona Ben-Haim

**Affiliations:** 1 Department of Nuclear Medicine & Biophysics, Hadassah-Hebrew University Medical Center, 91120 Jerusalem, Israel; 2 Department of Nuclear Medicine, Wolfson Medical Center, 58100 Holon, Israel; 3 Neuroendocrine Tumor Unit, ENETS Center of Excellence, Endocrinology and Metabolism Department, Hadassah-Hebrew University Medical Center, 91120 Jerusalem, Israel; 4 Oncology Department and Radiation Therapy Unit, Hadassah-Hebrew University Medical Center, 91120 Jerusalem, Israel; 5 Faculty of Medicine, Hebrew University of Jerusalem, 91120 Jerusalem, Israel; 6 Institute of Nuclear Medicine, University College London and UCL Hospitals NHS Trust, London, UK

**Keywords:** Q.Clear reconstruction, Reduced acquisition time or injected dose, Image quality, Visual evaluation, ^68^Ga-DOTATATE

## Abstract

**Background:**

Image quality and quantitative accuracy of positron emission tomography (PET) depend on several factors such as uptake time, scanner characteristics and image reconstruction methods. Ordered subset expectation maximization (OSEM) is considered the gold standard for image reconstruction. Penalized-likelihood estimation (PL) algorithms have been recently developed for PET reconstruction to improve quantitation accuracy while maintaining or even improving image quality. In PL algorithms, a regularization parameter *β* controls the penalization of relative differences between neighboring pixels and determines image characteristics. In the present study, we aim to compare the performance of Q.Clear (PL algorithm, GE Healthcare) and OSEM (3 iterations, 8 subsets, 6-mm post-processing filter) for ^68^Ga-DOTATATE (^68^Ga-DOTA) PET studies, both visually and quantitatively.

Thirty consecutive whole-body ^68^Ga-DOTA studies were included. The data were acquired in list mode and were reconstructed using 3D OSEM and Q.Clear with various values of *β* and various acquisition times per bed position (bp), thus generating images with reduced injected dose (1.5 min/bp: *β* = 300–1100; 1.0 min/bp: *β* = 600–1400 and 0.5 min/bp: *β* = 800–2200). An additional analysis adding *β* values up to 1500, 1700 and 3000 for 1.5, 1.0 and 0.5 min/bp, respectively, was performed for a random sample of 8 studies. Evaluation was performed using a phantom and clinical data. Two experienced nuclear medicine physicians blinded to the variables assessed the image quality visually.

**Results:**

Clinical images reconstructed with Q.Clear, set at 1.5, 1.0 and 0.5 min/bp using *β* = 1100, 1300 and 3000, respectively, resulted in images with noise equivalence to 3D OSEM (1.5 min/bp) with a mean increase in SUV_max_ of 14%, 13% and 4%, an increase in SNR of 30%, 24% and 10%, and an increase in SBR of 13%, 13% and 2%. Visual assessment yielded similar results for *β* values of 1100–1400 and 1300–1600 for 1.5 and 1.0 min/bp, respectively, although for 0.5 min/bp there was no significant improvement compared to OSEM.

**Conclusion:**

^68^Ga-DOTA reconstructions with Q.Clear, 1.5 and 1.0 min/bp, resulted in increased tumor SUV_max_ and in improved SNR and SBR at a similar level of noise compared to 3D OSEM. Q.Clear with *β* = 1300–1600 enables one-third reduction of acquisition time or injected dose, with similar image quality compared to 3D OSEM.

## Background

Positron emission tomography (PET) computed tomography (CT) with ^68^Ga-DOTATATE (^68^Ga-DOTA) is widely used for imaging of neuroendocrine tumors with significant roles in staging, assessment of somatostatin receptor status and decision-making regarding therapy regimens. The current procedure guidelines for PET/CT tumor imaging with ^68^Ga-DOTA-conjugated peptides [1] recommends an injected activity ranging between 100 and 200 MBq depending essentially on the characteristics of the PET tomograph. In order to reduce patient dose, and considering that ^68^Ga-DOTA availability is limited by ^68^Ge/^68^Ga generator capacity, reduced injected doses with preserved image quality should be investigated, with the ultimate aim of defining the most appropriately low level of injected activities.

Image quality and quantitative accuracy of PET studies are highly influenced by several factors such as injected activity, uptake time, scanner characteristics and image reconstruction methods. Currently, statistical iterative reconstruction methods are the most widely used image reconstruction methods [2] and the ordered subset expectation maximization (OSEM) statistical method is the gold standard. OSEM algorithms approach the acquired image by successive updated approximations, repeated until the difference between the projections of the reconstructed image and the actually recorded one falls below a specific level. The major drawback of OSEM is that the iteration process has to be stopped before convergence in order to avoid image degradation due to excessive noise. This early stop leads to a bias in the final image estimate toward the initial image and to a decrease in contrast recovery (CR), signal-to-noise ratio (SNR) and image quality, which is partly accountable to the ineffective convergence of the algorithm. Moreover, the reconstructed images are typically post-filtered with a Gaussian low-pass filter in order to reduce background noise and to improve the signal-to-noise ratio (SNR) of the image with better contrast [3]. The post-filter is also used to remove Gibbs artifact at edges when OSEM with Point Spread Function (PSF)-based reconstruction (resolution modeling) is used [4].

Penalized-likelihood estimation (PL) reconstruction algorithms have been recently developed and clinically implemented to improve quantitation accuracy, while maintaining or even improving image quality. PL algorithms allow for fully convergent iterative reconstruction, leading to higher image contrast than for OSEM while limiting noise [5]. Instead of the Gaussian kernel filter, image characteristics are determined by a regularization *β* parameter which controls the penalization of relative differences between neighboring pixels [6]. Therefore, with PL algorithms, the sole *β-*positive regularization parameter controls the trade-off between noise level and resolution, as opposed to several iterations, subsets and post-filter with OSEM. Moreover, with PL algorithms, excessive smoothing over large edges and Gibbs artifacts from PSF modeling are avoided [6].

Q.Clear is the commercially available version of the PL algorithm introduced by General Electric (GE) Healthcare. There is no one optimal penalization *β* factor but its value depends on different parameters such as the radiopharmaceutical, the injected activity, the acquisition time per bed position (bp), the PET scanner and the image reconstruction algorithm. In both phantom and clinical ^18^F-FDG studies, Q.Clear has been shown to provide better quantitation accuracy and image quality than OSEM [7–10]. A study using the same PET/CT scanner (General Electric, Discovery MI) and comparing Q.Clear to OSEM (4 iterations, 16 subsets, 2-mm post-filter) [11] showed that for ^18^F-FDG examinations, the optimal *β* ranges between 500 and 600 when using a (administered activity (MBq/kg) × acquisition time (min/bp)) product of 6. A recent study [12] showed that using a *β* factor between 300 and 450, Q.Clear is superior to OSEM (4 iterations, 16 subsets, 2-mm post-filter) including time-of-flight (TOF) information and PSF modeling, in terms of CR and SNR. For ^68^Ga-DOTA, one preliminary study by Lantos et al. [13] in 10 patients suggested using a *β* factor between 350 and 450 in clinical practice for all the studied radiopharmaceuticals while the study conducted by ter Voert et al. [14] concluded that, for ^68^Ga-PSMA, a *β* value between 400 and 550 could be optimal. A recent study conducted by Lindström et al. [15] on thirteen ^68^Ga-DOTATOC studies concluded that *β* values equal to or higher than 400 result in noise levels equal to or lower than those of OSEM (3 iterations, 16 subsets, 5-mm post-filter) with improved SNR and SBR.

In the present study, we aimed to evaluate quantitatively and qualitatively the performance of Q.Clear with full and reduced acquisition time or injected activity, compared to our local optimized OSEM + TOF + PSF (3 iterations, 8 subsets, 6-mm post-filter) and to the manufacturer’s recommendation OSEM + TOF + PSF (3 iterations, 16 subsets, 5-mm post-filter) with or without time or dose reduction for whole-body ^68^Ga-DOTA examinations. We defined an optimal *β* value for the standard acquisition time, and we investigated optimal *β* values leading to similar or even superior image quality while the acquisition time or injected activity had been reduced. To achieve this goal, Q.Clear and OSEM acquisitions were compared by quantitative evaluation of phantom acquisitions and clinical studies, as well as by qualitative assessments.

## Methods

### Patient population

Between March 17, 2019, and June 19, 2019, 65 consecutive patients underwent ^68^Ga-DOTA PET/CT scans at our institution. Inclusion criteria for this study were (a) images were acquired on General Electric (GE) Healthcare Discovery MI PET/CT scanner (Milwaukee, WI, USA) and (b) at least one focus of pathological ^68^Ga-DOTA uptake was noted on the PET/CT study.

Of the 65 patients, 35 patients were excluded from the study, 21 patients with normal studies without focus of pathological uptake and 14 patients performed ^68^Ga-DOTA PET/CT on another PET/CT system (Discovery MI-DR, GE Healthcare) at our institution. The remaining 30 patients (19 men, 11 women; mean ± SD, 58 ± 19 years old; range 11–83 years) were included in this single-center retrospective study.

### Data acquisition

All studies were performed on a Discovery MI PET/CT (GE Healthcare). The system combines a 128-slice computed tomography (CT) system and a 4-ring PET system with LightBurst digital detectors providing a 20-cm axial field-of-view and a 70-cm transaxial field-of-view. The system is TOF-capable with a timing resolution of 377 ps [16].

#### Phantom acquisition

The National Electrical Manufacturers Association (NEMA) IEC image quality body phantom (IQBP) (Model PET/IEC-BODY/P) [17] was used to provide an overall assessment of the imaging capabilities of the system in different conditions. The phantom contains spheres with an internal diameter of 10, 13, 17, 22, 28 and 37 mm and a 50-mm diameter cylindrical insert mounted in its center. All the spheres were filled with radioactive material (^68^Ga), and lung insert provided with the phantom was filled with low-density material (polystyrene) and water. The phantom was filled to reach a target-to-background ratio of 4:1. The background region and spheres contained a ^68^Ga activity concentration of 2.48 kBq/mL and 9.92 kBq/mL, respectively, at the time of acquisition. The phantom images were acquired in list mode with an acquisition time of 3.0 min/bp.

#### Clinical images

^68^Ga-DOTA was injected intravenously following an administration protocol of 2 MBq/kg (minimum–maximum activity: 100–250 Mbq). The mean administered activity was 160.3 ± 32.0 MBq (range, 103.6–247.9 MBq). The PET acquisition started at a mean of 68 ± 10 min (range, 53–91 min) after tracer injection. All PET studies were performed from the proximal femur to the base of the skull (six–eight bed positions) and were acquired in list-mode with an acquisition time of 1.5 min/bp. Patients’ characteristics, injected dose and uptake time are summarized in Table 1.

**Table 1:** Demographic data and patient characteristics

Pt no.	Age (years)	Body weight (kg)	Dose (MBq)	Uptake time (min)	Primary tumor	Primary tumor site	Sites of metastases
1	78	59	170.2	74	Small bowel NET	Resected	Liver, LN above and below the diaphragm
2	73	100	155.4	69	Pheochromocytoma	Local recurrence in right adrenal bed	Retroperitoneal LN, omentum
3	68	97	199.8	58	Lung NET	Resected	Mediastinal LN, bones
4	46	71	188.7	61	Pancreatic NET	Pancreas	Mesenteric, retroperitoneal LN, liver, bones
5	28	70	144.3	76	Pancreatic NET	Pancreas	(nonconclusive bone focus, resolved on FUP)
6	11	43	155.4	62	Lung carcinoid	Lung mass	Mediastinal LN
7	64	53	133.2	76	Pancreatic NET	Local recurrence in surgical bed	Liver, Liver hilum LN, bones
8	66	68	159.1	70	Lung NET	Lung several foci	Mediastinal and right hilar LN
9	60	59	159.1	54	Glucagonoma	Local recurrence in surgical bed	Mesenteric LN, mediastinal LN, bones
10	83	65	159.1	81	Pancreatic NET	Pancreas	Right iliac crest and mediastinal LN
11	79	60	159.1	57	Unknown origin	Unknown	Liver, bone
12	27	53	173.9	62	Bronchial carcinoid	Resected	Bones, soft tissue
13	49	65	122.1	65	Pancreatic NET	Pancreas	Liver
14	66	59	136.9	74	Pancreatic NET	Resected , local recurrence	Liver
15	67	75	162.8	74	Small bowel NET	Resected	Pancreas, liver
16	70	119	203.5	63	Appendiceal carcinoid	Resected	Liver
17	71	59	148	78	Pancreatic NET	Pancreas	Liver
18	73	110	247.9	79	Pancreatic NET	Pancreas	N/A
19	13	40	129.5	53	Insulinoma	Pancreas	Liver, retroperitoneal LN
20	38	83	111.0	88	Pancreatic NET	Pancreas	LN above and below the diaphragm
21	75	72	122.1	91	Lung NET	Lung	Brain
22	72	88	140.6	58	Small bowel NET	Resected	Liver, omentum
23	59	46	166.5	61	Small bowel NET	Resected	Liver hilum LN
24	56	71	103.6	73	Pancreatic NET	Resected	Liver, bones
25	38	65	162.7	72	Lung NET	Lung	N/A
26	60	63	185	57	Small bowel NET	Resected	Liver
27	65	80	144.3	78	Medullary Thyroid Ca	Left lobe resected	Surgical bed
28	42	72	148.2	59	Small bowel NET	Resected	Liver, mesenteric LN
29	74	66	185.0	69	Paraganglioma	Resected	Local recurrence (brain), bone
30	70	84	233.1	58	Unknown origin	Unknown	Bones, right hilum
Mean ± SD	58 ± 19	71 ± 18	160.3 ± 32.0	68 ± 10	–	–	–

*NET* neuroendocrine tumor, *LN* lymph node, *FUP* follow-up plan

### Image reconstruction

Phantom and clinical images were first reconstructed with 1.5 min/bp and using the GE VUE Point FX-S algorithm (VPFX-S), a 3D maximum likelihood ordered subset expectation maximization (3D OSEM) image reconstruction algorithm using TOF information and PSF modeling with 3 iterations, 8 subsets and 6-mm post-processing filter. These settings have been adjusted at the installation of the system by the local manufacturer field engineer according to the visual evaluation of ^68^Ga-DOTA PET images done by experienced physicians. The corresponding reconstructed images are defined as Hadassah OSEM reconstruction thereafter.

In addition, data were reconstructed using the Q.Clear algorithm with different values of the penalization factor *β* and with 1.5, 1.0 and 0.5 min/bp acquisitions. The 1.5, 1.0 and 0.5 min/bp acquisitions were used to simulate standard, two-thirds and one-third acquisitions (time or injected dose). Images were reconstructed in a first time with *β* = 300, 400, 500, 600, 700, 800, 1000 and 1100 for the 1.5-min/bp acquisition, *β* = 600, 700, 800, 1000, 1100, 1200 and 1300 for the 1.0 min/bp acquisition and *β* = 800, 1000, 1200, 1300, 1400, 1500, 1600, 1800, 2000 and 2200 for the 0.5 min/bp acquisition. These values were chosen following an initial subjective visual assessment of clinical images performed by one of the authors (AC, who was not involved in the blinded visual assessment of the studies).

After a first quantitative analysis and visual assessment of the images reconstructed with the previous parameters, it seemed that higher *β* values are to be included in the analysis. Thus, an additional analysis adding *β* values of 1200, 1300, 1400, 1500 for 1.5 min/bp, 1400, 1500, 1600, 1700 for 1.0 min/bp and 2400, 2600, 2800, 3000 for 0.5 min/bp was performed for a random group of 8 patients. OSEM reconstruction recommended by the manufacturer to be used in clinical setting (3 iterations, 16 subsets, 5-mm post-processing filter) [18, 19] was also added to this additional analysis and defined as GE OSEM reconstruction.

All data were corrected for scatter, random events, dead time and attenuation (using CT).

### Image analysis

Images were analyzed as detailed below and previously proposed by Lindström et al. [19].

#### Phantom data

Background variability (BV) and contrast-to-noise ratio (CNR) were calculated and compared. BV was defined as the SD of the activity concentration in large ROIs (about 4 cm^2^) located away from the axial plane containing the sphere centers, divided by the mean activity concentration in these background ROIs. CNR was calculated as contrast recovery (CR) divided by BV as follows:

1 $$ {\displaystyle \begin{array}{c}\mathrm{CNR}=\mathrm{CR}/\mathrm{BV}\\ {}\mathrm{where}\kern0.15cm \mathrm{CR}=\frac{\frac{{\mathrm{C}}_{\mathrm{H}}}{{\mathrm{C}}_{\mathrm{B}}}-1}{\frac{{\mathrm{a}}_{\mathrm{H}}}{{\mathrm{a}}_{\mathrm{B}}}-1}\end{array}} $$

with *C*_H_ and *C*_B_, counts and a_H_ and a_B_, activities in hot spheres and background ROIs, respectively. Image analysis was done on a GE Healthcare Advantage Workstation (AW 3.2 Ext. 3.2, 2019).

#### Clinical images

Level of noise, signal-to-noise ratio (SNR) and signal-to-background ratio (SBR) were calculated and compared. Level of noise was defined as SUV_std_ of a large spherical VOI in normal liver normalized to SUV_mean_ of the same VOI. SNR was calculated as lesion SUV_max_ divided by noise level. SBR was defined as lesion SUV_max_ divided by SUV_mean_ of the normal liver VOI. For this analysis, up to three lesions per study (for a total of 75 in the first analysis and 19 in the additional one) were delineated on the AW Workstation using a 41% SUV_max_ threshold. Lesion VOIs were first built on the Hadassah OSEM images, and bookmarks containing the location of these lesions were used to propagate and build new VOIs on reconstructed images with 41% thresholding. Also, lesions SUV_max_ values obtained for Hadassah OSEM and the reconstructed algorithms leading to similar level of noise were also analyzed and compared. The correlation of the SNR and SBR with the lesion size, penalization factor value, injected activity and patient weight has been evaluated for the three acquisition times.

#### Blinded visual assessment

In the first analysis, whether for the standard acquisition or for each of the simulated reduced injected activity studies, the five Q.Clear reconstructions leading to the best results in phantom and clinical images evaluations were visually compared with the Hadassah OSEM reconstruction by two blinded experienced nuclear medicine physicians (EG and SBH). A total of 90 image sets were assessed; every set consisted of 5 different reconstructions for 1.5, 1.0 and 0.5 min/bp, for each of the 30 patients included in the study. For the additional analysis, the four additional Q.Clear reconstructions and the manufacturer reference GE OSEM, were visually compared with the Hadassah OSEM reconstruction by an experienced reader (SBH). All data were anonymized regarding the reconstruction method, and numbers were randomly assigned. PET datasets were rated on a 4-point scale (1 = very poor/nondiagnostic; 2 = poor; 3 = good and 4 = very good) for contrast, sharpness, noise, liver homogeneity, tumor detectability and overall image quality.

### Statistical analysis

The mean values for each of the rated parameters by the two readers and the mean values of all scores were summarized. A non-parametric test, Friedman test, for multiple comparisons was performed to evaluate the differences between the various image reconstruction algorithms. When necessary, a correction for ties was applied [20]. When Friedman test indicated significance (*p* < 0.05), it was followed by post-hoc pairwise comparisons (between a given algorithm and Hadassah OSEM) according to Conover [21], with Bonferroni adjustment. Bonferroni corrected *p* values lower than 0.05 were considered statistically significant. For the quantitative analysis, algorithms with significant Bonferroni corrected *p* values for SNR, SBR and noise were considered to outperform Hadassah OSEM. For the visual analysis, algorithms leading to significant Bonferroni corrected *p* values for the mean of all the rated parameters were considered superior to Hadassah OSEM.

## Results

### Phantom studies

**Fig. 1:**
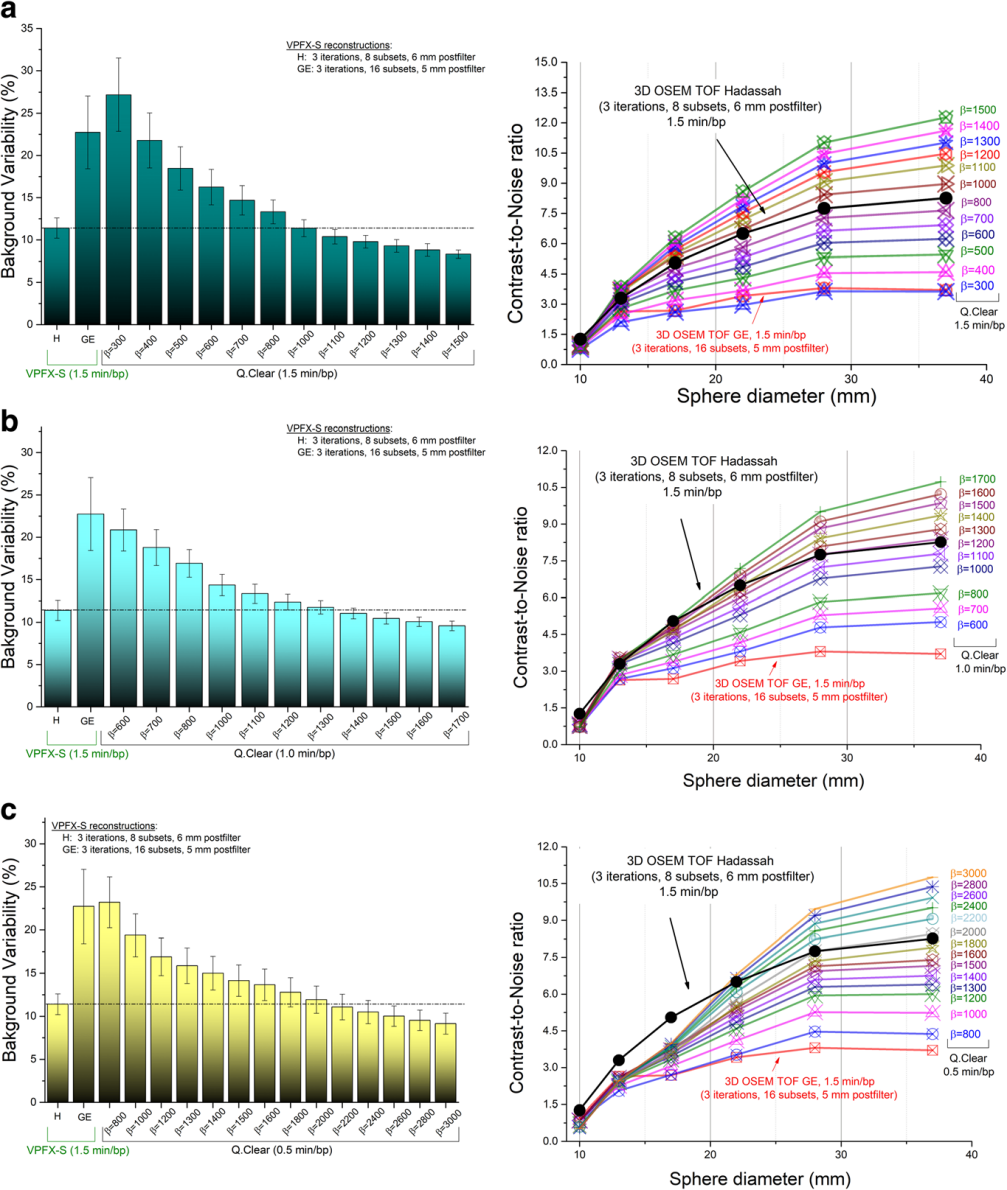
NEMA IEC image quality body phantom *(i)* background variability (SD of the activity concentration in large ROIs of about 4 cm^2^) and *(ii)* contrast-to-noise ratio using Hadassah (H) and manufacturer recommendation (GE) OSEM+TOF+PSF reconstruction algorithms and, Q.Clear with different values of *β* and (**a**) 1.5 min/bp, (**b**) 1.0 min/bp and (**c**) 0.5 min/bp. The phantom background region and spheres contained a ^68^Ga activity concentration of 2.48 kBq/mL and 9.92 kBq/mL (4:1 sphere-to-background ratio), respectively, at the time of acquisition

Background variability and contrast-to-noise ratio obtained from images reconstructed with Hadassah OSEM, the manufacturer (GE OSEM) recommendation (OSEM + TOF + PSF, 1.5 min/bp) and Q.Clear with different values of the penalization factor *β* and acquisition times of 1.5, 1.0 and 0.5 min/bp are shown in Fig. 1a–c. The Q.Clear algorithm with *β* ≥ 1000 for 1.5 min/bp, *β ≥* 1400 for 1.0 min/bp and *β* ≥ 2200 for 0.5 min/bp allowed for similar or improved BV values compared to the Hadassah reconstruction method. Similarly, CNR values obtained with Q.Clear are higher than those with Hadassah OSEM when using *β* ≥ 1000 for 1.5 min/bp and *β* ≥ 1300–1500 for 1.0 min/bp. For 0.5 min/bp, improved CNR results are reached for *β* ≥ 2200 for the large 28- and 37-mm spheres while for the 17- and 13-mm spheres CNR values were about 25% lower compared to the Hadassah OSEM reconstruction method. Of note, for all reconstructions, the standard Hadassah OSEM reconstruction led to better CNR for the smallest 10-mm diameter sphere.

Comparing Q.Clear reconstructions to the reconstruction recommended by the manufacturer (GE OSEM), penalization factors of *β* ≥ 400 for 1.5 min/bp and of *β* ≥ 600 allowed to obtain a better BV and CNR. For 0.5 min/bp, Q.Clear reconstructions with *β* values higher than 1000 outperforms the BV obtained when using the manufacturer recommendation and *β* ≥ 800 allowed to obtain higher or similar CNR values for all the spheres except for the two smallest ones.

**Fig. 2:**
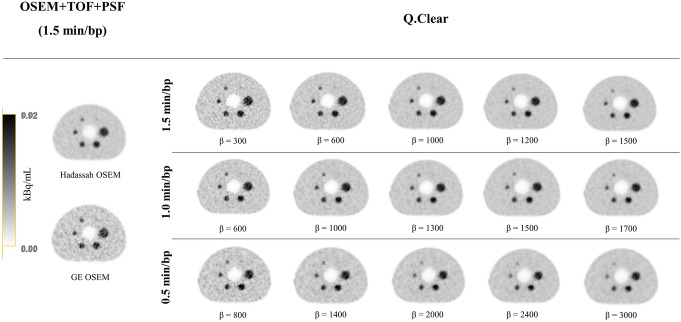
Central slice of the NEMA IEC image quality body phantom reconstructed with the Hadassah and GE OSEM reconstruction algorithms (1.5 min/bp) and with Q.Clear for different values of *β* and acquisition time per bed position (1.5, 1.0 and 0.5 min/bp). The phantom background region and spheres contained a ^68^Ga activity concentration of 2.48 kBq/mL and 9.92 kBq/mL (4:1 sphere-to-background ratio), respectively, at the time of acquisition. The gray scale represents the activity concentration in kBq/mL for all phatom images

Figure 2 presents the transverse views of the phantom acquisitions, reconstructed with the Hadassah OSEM and GE OSEM algorithms with 1.5 min/bp and with Q.Clear for different values of *β* and different acquisition times per bed position (1.5, 1.0 and 0.5 min/bp). The images demonstrate better BV and CR with increasing *β* factor for all the acquisition times.

### Clinical images

**Fig. 3:**
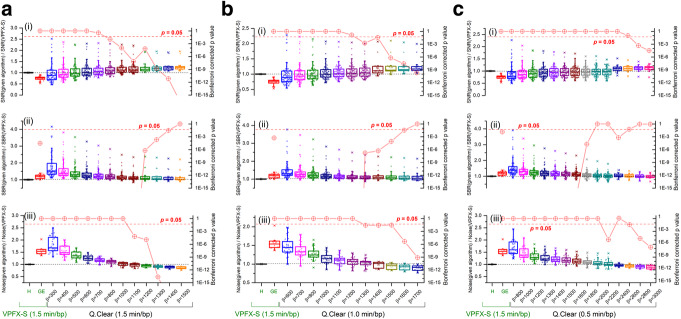
Box plots of (i) SNR, (ii) SBR and (iii) noise level values calculated from studies reconstructed using Q.Clear with various *β* and **a** 1.5 min/bp, **b** 1.0 min/bp and **c** 0.5 min/bp and GE OSEM (1.5 min/bp), normalized to the values obtained using the Hadassah OSEM reconstruction algorithm with 1.5 min/bp (left *Y*-axis). The upper and lower part of the box represent the upper and lower quartile, respectively. The line and the square in the box stand for the median and the mean values, respectively. The Bonferroni corrected *p* values for a given algorithm compared to Hadassah OSEM are shown in red circles (right *Y*-axis)

Figure 3a–c presents the SNR, SBR and noise level values calculated from clinical studies reconstructed using Q.Clear with 1.5, 1.0 and 0.5 min/bp, respectively, and normalized to Hadassah OSEM SNR, SBR and noise level values. The Bonferroni corrected *p* values for a given algorithm compared to Hadassah OSEM are also shown in Fig. 3 (red circles, right *Y*-axis). A total of 75 lesions for the first analysis and 19 for the additional one per reconstruction were used for comparative analysis of the SNR and SBR obtained using Q.Clear, with different *β* values and acquisition times, to those obtained with the Hadassah OSEM reconstruction and 1.5 min/bp. As described above, a single large normal liver VOI was used in each study for noise level comparison.

Regardless of the acquisition time per bed position, the choice of the penalization factor influenced the different parameters, resulting in improvement of the noise level and the SNR and degradation of SBR for increasing *β* values. For the smallest *β* values used in this study (i.e. *β* = 300 for 1.5 min/bp, *β* = 600 for 1.0 min/bp and *β* = 800 for 0.5 min/bp), the noise level increased by about 85%, 50% and 65% on average, respectively, compared to the standard Hadassah OSEM reconstruction method. However, the noise level was lower than the standard by 15% using *β* = 1500 with 1.5 min/bp and 10% using *β* = 1700 with 1.0 min/bp or *β* = 3000 with 0.5 min/bp. For the latter, the SNR increased by 30%, 24% and 12%, respectively. The SBR was also higher than the Hadassah standard with a mean increase of 13% for 1.5 and 1.0 min/bp and lower by 2% in average for 0.5 min/bp. The SUV_max_ increased by 14%, 13% and 4% on average, respectively. The statistical results show that a *β* = 1100–1400 for 1.5 min/bp outperforms Hadassah OSEM in terms of SNR, SBR and noise level with Bonferroni corrected *p* < 0.05 (Fig. 3a). For 1.0 min/bp, *β* = 1300–1600 allowed to obtain a better SNR, SBR and noise level (Bonferroni corrected *p* < 0.05) (Fig. 3b). For 0.5 min/bp, using *β* = 2800–3000, only the SNR and noise are improved (Fig. 3c).

Comparison of GE OSEM and Q.Clear reconstructions shows that for 1.5 min/bp a *β* value of 500 is sufficient to outperform OSEM in terms of SNR, SBR and noise level (Bonferroni corrected *p* < 0.05 for all) with an average improvement of 30%, 28% and 20%, respectively. For 1.0 min/bp, Q.Clear with *β* = 700–800 is also better than GE OSEM with SNR and SBR increasing, respectively, by 27% and 14% and noise level decreasing by 25% on average. The reduction of the acquisition time to 0.5 min/bp with Q.Clear allowed to obtain improved SNR, SBR and noise levels but improvements are not significant. Compared to Hadassah OSEM, GE OSEM has a lower SNR by 25%, a higher noise level by 60% and an improved SBR by 18%.

**Fig. 4:**
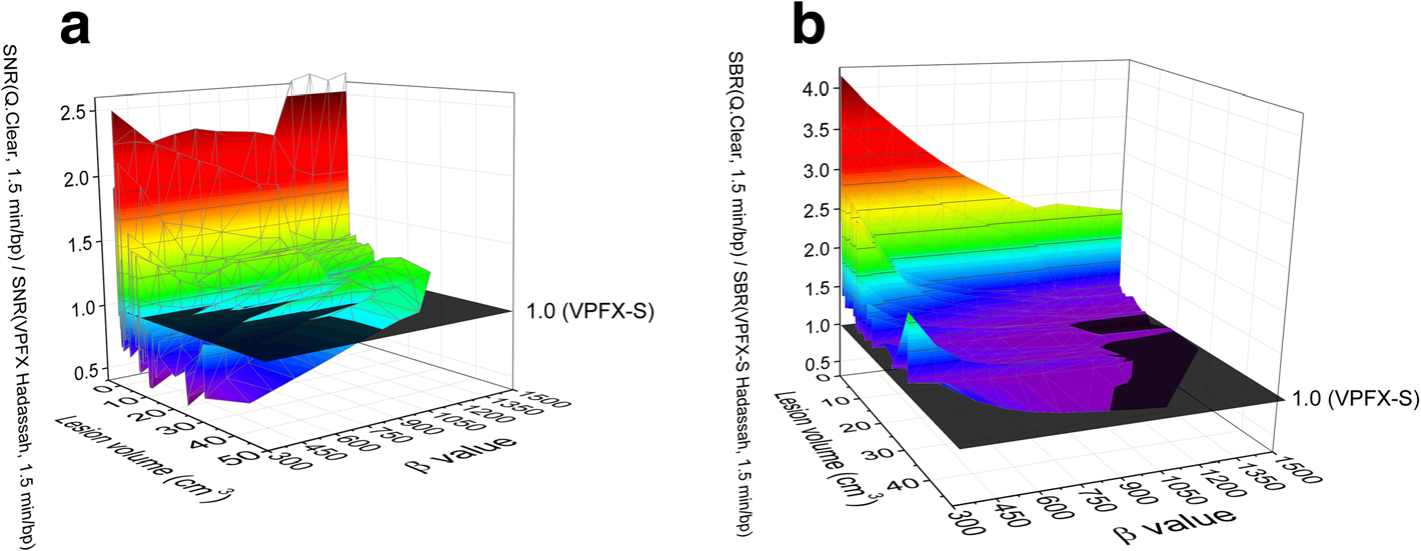
Improvement of **a** SNR and **b** SBR using Q.Clear with 1.5 min/bp over Hadassah OSEM as a function of the *β* penalization factor and tumor lesion volume

The SNR and SBR improvements obtained with Q.Clear in comparison to the Hadassah OSEM reconstruction were assessed as a function of the lesion size, injected activity and patient weight for 1.5, 1.0 and 0.5 min/bp. There was no correlation between the SNR or SBR improvement and the injected activity per kilogram (MBq/kg) or uptake time. The improvement in SNR or SBR was stable despite variation of these parameters. However, there was a correlation with the lesion volume, foci with volumes lower than 1–2 cm^3^ showing the highest improvement in both SNR and SBR (corresponding to the outliers observed in Fig. 3). For these small lesions, an increase of *β* resulted in a decrease in the improvement of the SNR whereas SNR improved with an increase of *β* in larger lesions. Also, for lesions larger than 10 cm^3^, the improvement in SNR for a given *β* remained stable as a function of the lesion volume. Figure 4a, b presents the SNR and SBR improvement using Q.Clear with 1.5 min/bp as a function of the lesion size and the *β* value, respectively.

### Visual assessment

**Fig. 5:**
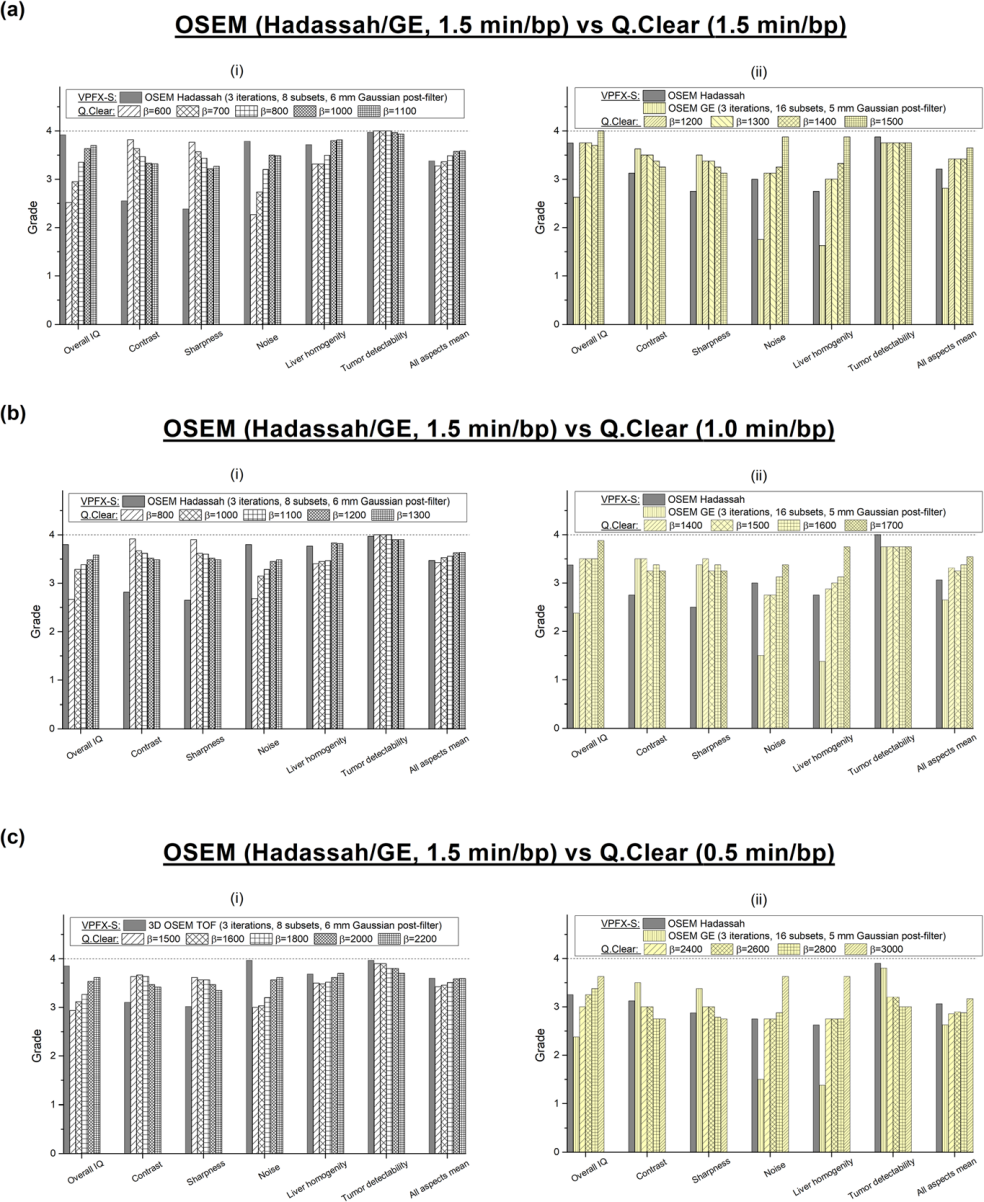
Mean of visual assessment (overall image quality, contrast, sharpness, noise level, liver homogeneity, tumor detectability and mean of all rated aspects) for Q.Clear reconstructions with different *β* values and **a** 1.5 min/bp, **b** 1.0 min/bp and **c** 0.5 min/bp and Hadassah and GE OSEM recontructions (1.5 min/bp) following a 4-point scale (1, very poor/nondiagnostic; 2, poor; 3, good and 4, very good). The first analysis (i) included 30 patients against 8 patients for the addiotnal one (ii)

Five Q.Clear reconstructions (i.e. *β* = 600–1100 for 1.5 min/bp, *β* = 800–1300 for 1.0 min/bp, *β* = 1500–2200 for 0.5 min/bp) were compared with the Hadassah OSEM reconstruction at first stage (Fig. 5 (i)), and additional *β* values (i.e. *β* = 1200–1500 for 1.5 min/bp, *β* = 1400–1700 for 1.0 min/bp, *β* = 2400–3000 for 0.5 min/bp) and GE OSEM were then added to the analysis (Fig. 5 (ii)). Hadassah OSEM was graded with each acquisition time and for both analyses. Figure 5a–c shows the mean grades given to the following image quality parameters: overall image quality, contrast, sharpness, noise level, liver homogeneity and tumor detectability, for OSEM (Hadassah and GE), 1.5 min/bp, and Q.Clear reconstructions with 1.5, 1.0 and 0.5 min/bp, respectively. Also shown are the mean values of all aspect scores.

**Fig. 6:**
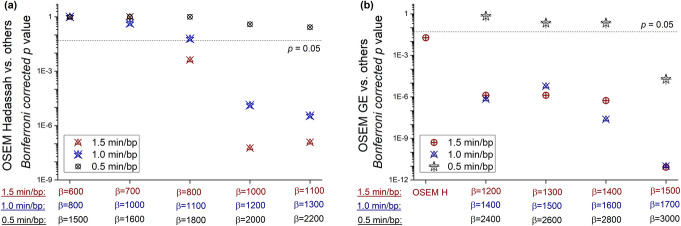
*p* values (non-parametric one-tail sign test) of the improvement in the mean score of all rated aspects between reconstructions using Q.Clear and **a** Hadassah OSEM or **b** GE OSEM

In the first analysis, for the full-time acquisition of 1.5 min/bp, Q.Clear reconstruction with *β* = 1100 yielded the highest mean grade of all parameters with a score of 3.59 and Bonferroni corrected *p* < 10^−7^ (Fig. 6). Reconstructions with *β* = 800 and 1000 also ranked better than Hadassah OSEM with mean scores of 3.49 and 3.57 (Bonferroni corrected *p* < 0.05), respectively, compared to 3.38 for the standard. Similarly, 1.0-min/bp images reconstructed with Q.Clear and *β* = 1200 and 1300 scored better than Hadassah OSEM with 1.5 min/bp with Bonferroni corrected *p* values of 1∙10^−5^ and 4∙10^−6^, respectively. However, for 0.5 min/bp, Q.Clear scored 3.60 for *β* = 2200 compared to 3.61 for Hadassah with 1.5 min/bp (corrected *p* = 0.26).

**Fig. 7:**
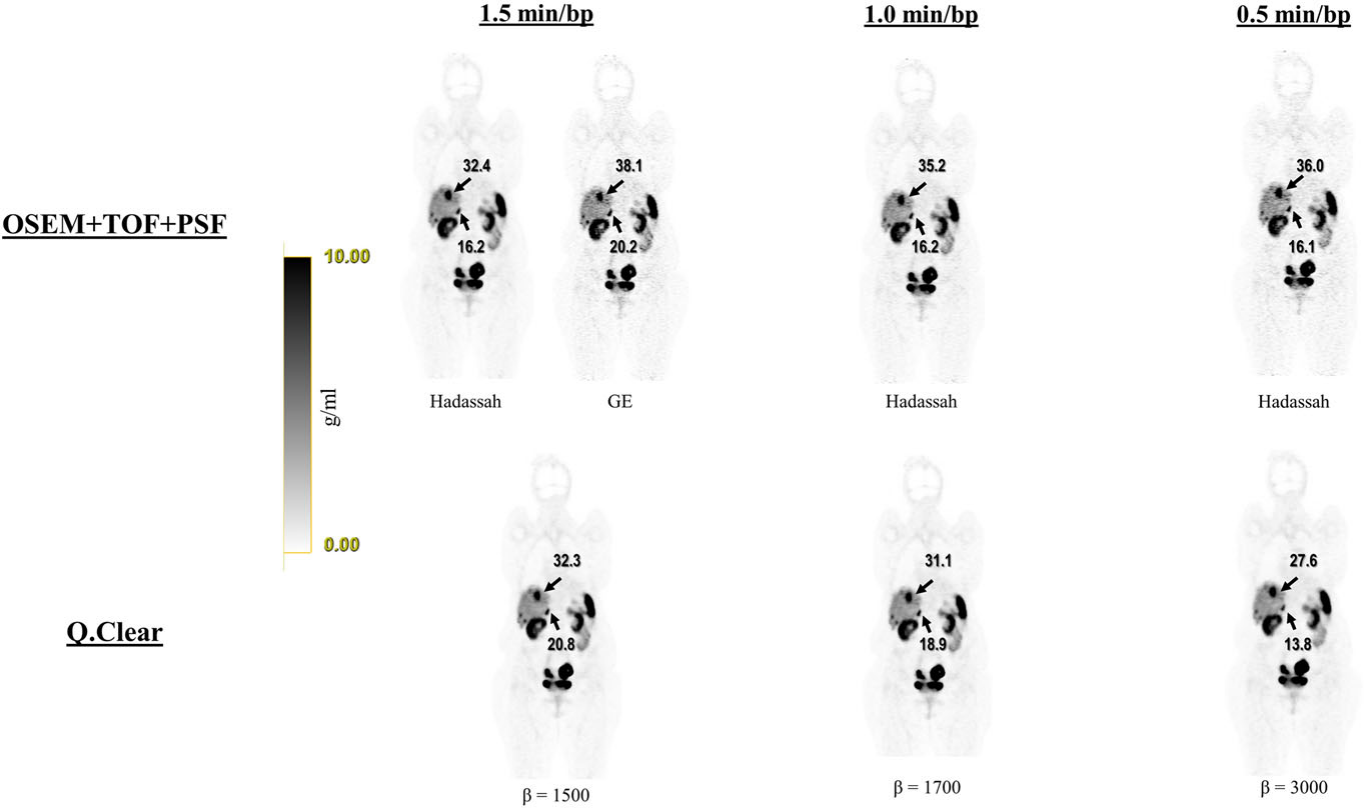
Representative coronal ^68^Ga-DOTA images of patient no. 1 reconstructed with Hadassah (1.5, 1.0 and 0.5 min/bp) and manufacturer recommendation GE OSEM (1.5 min/bp) and, Q.Clear, for 1.5, 1.0 and 0.5 min/bp and *β* = 1500, 1700 and 3000, respectively. The image quality of Q.Clear with 1.0 and 0.5 min/bp is better compared to Hadassah OSEM with the same acquisition time and is similar when using 1.5 min/bp. The image quality seems better with Q.Clear than for GE OSEM for all acquisition times. SUV_max_ uptake values (g/ml) obtained with the different reconstruction methods for two lesions are indicated by arrows. The gray scales next to the images represents the corresponding SUV scale in g/ml

**Fig. 8:**
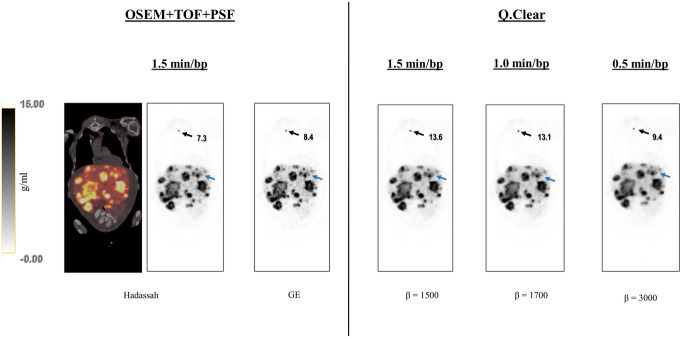
Representative coronal ^68^Ga-DOTA images of patient no. 7 reconstructed with Hadassah and GE OSEM (1.5 min/bp) and Q.Clear with 1.5, 1.0 and 0.5 min/bp. Tumor detectability, contrast and contrast using Q.Clear 1.5 min/bp (*β* = 1500) and 1.0 min/bp (*β* = 1700) was better than those using OSEM with 1.5 min/bp, but in using Q.Clear with 0.5 min/bp (*β* = 3000) some lesions were missed (blue arrow for instance). Sternum lesion SUV_max_ uptake value (g/ml) obtained with the different reconstruction methods is indicated by black arrows. The gray scale next to the images represents the corresponding SUV scale in g/ml

The second analysis extended the *β* values and added GE OSEM for comparison in 8 studies. It is noteworthy that higher *β* values improved all the variables except for tumor detectability. Reconstructions with *β* = 1500 and 1700 for 1.5 and 1.0 min/bp ranked better than Hadassah OSEM with mean scores of 3.64 and 3.54 compared to 3.21 and 3.07, respectively (all with Bonferroni corrected *p* < 0.05). The reconstruction recommended by the manufacturer, GE OSEM, ranked below Hadassah OSEM for all the aspects except contrast and sharpness. Comparing GE OSEM to Q.Clear, *β* of 1200 and 1400 for 1.5 and 1.0 min/bp, respectively, allowed to obtain higher mean grades with Bonferroni corrected *p* < 0.05 (Fig. 6b). Of note, for all *β* values assessed here, regardless of acquisition time, the Q.Clear algorithm presented a definite advantage in terms of contrast and sharpness compared to Hadassah OSEM (Figs. 7 and 8). On the other hand, tumor detectability for small lesions has been degraded using Q.Clear with 0.5 min/bp as shown in Fig. 8. For 1.5 and 1.0 min/bp, tumor detectability was conserved or even improved for the lowest *β* values, although the differences were not statistically significant.

## Discussion

The present study focused on determining optimal *β* values for ^68^Ga-DOTA studies reconstructed with our local optimized OSEM reconstruction and when the acquisition time or injected activity is reduced by one- and two-thirds. The study included also the OSEM reconstruction recommended by the vendor and showed that optimal *β* values are very dependent of the OSEM reconstruction used as reference.

Phantom evaluation allowed us to determine that using the Q.Clear algorithm with *β* ≥ 1000 for 1.5 min/bp and *β* ≥ 1400 for 1.0 min/bp similar or improved BV and CNR values are obtained compared to Hadassah OSEM. However, the results showed that for 0.5 min/bp, improved CNR results are reached for *β* = 2200 for the large spheres but reduced by about 25% for the small volumes. This last observation can be related to the visualization assessment results obtained for 0.5 min/bp where the tumor detectability was greatly degraded in comparison to Hadassah OSEM. For 1.5 and 1.0 min/bp, tumor detectability seems to be preserved with Q.Clear on the first visual evaluation, although phantom images suggested a detectability degradation for 10-mm lesions with increasing *β* values. Evaluation of noise, SNR and SBR on clinical studies and visualization assessment results suggested that optimal *β* values would be of 1100–1400 for 1.5 min/bp and 1300–1600 for 1.0 min/bp. However, for 0.5 min/bp acquisitions, Q.Clear did not demonstrate a clear advantage compared to Hadassah OSEM with 1.5 min/bp. An acquisition time of 0.5 min is perhaps too short in order to get reliable data. Indeed, image quality was improved with high *β* values, but it seems that the in-data is too noisy to obtain a reliable outcome.

Clinical images were also evaluated to assess the improvement in SNR and SBR using Q.Clear with different *β* values and different acquisition times compared to Hadassah OSEM with 1.5 min/bp. The box plots showed mostly a positive skew distribution indicating frequent SNR or SBR values in the lower part of the box and few high values with some outliers (Fig. 3). The outliers had up to 2.5 times SNR and 4 times SBR improvement using Q.Clear compared to OSEM. The analysis of the improvement in SNR and SBR using Q.Clear as a function of the lesion size showed the highest improvement in lesions with volumes lower than 1–2 cm^3^. This is in accordance with previous observation by Lindström et al. [19] where the relative difference in SUV_max_ between Q.Clear and 3D OSEM was larger for smaller lesions.

Our standard Hadassah OSEM reconstruction was also compared to the OSEM reconstruction recommended by the manufacturer, GE OSEM. From phantom and image analysis, it is clear that Hadassah OSEM shows better BV, CNR, SNR and noise level. The only point where GE OSEM is better is SBR. Similarly, the visual assessment ranked GE OSEM at a lower level than Hadassah OSEM for overall image quality, noise and tumor detectability. The higher level of noise in GE OSEM may not enable visualization of small lesions, leading consequently to reduced tumor detectability. These differences cause extremely different *β* values to outperform the corresponding OSEM algorithm (*β* ≈ 500 for GE OSEM vs *β* ≈ 1200 for Hadassah OSEM).

Previous studies also investigated optimal values of *β* for ^68^Ga tracers. In a preliminary study conducted by Lantos et al. [13], the authors suggested to use a *β* value of 350–450 in clinical practice for all radiopharmaceuticals, including ^68^Ga-DOTA. The much higher *β* values obtained in our study can be due to different reasons. First of all, Lantos et al. compared the SUV_max_ of the smallest lesion of 10 patients using different reconstructions (3D OSEM vs PL with beta = 250, 350 and 450). As shown in the present study (Fig. 4) and by other authors [14, 19], the smaller the size of the lesions the better the improvement in SNR compared to OSEM. Taking into account only the smallest lesion in their study possibly shifted the optimal *β* values downwards. Also, higher than *β* = 450 values were not investigated and were not in the scope of their work. Moreover, the authors did not specify the acquisition time per bed position or the injected activity for the clinical studies considered. Finally, the OSEM reconstruction parameters—number of iterations, subsets and the filter—are not detailed. Therefore, a direct comparison between present results and those of [13] is challenging. Recently Lindström et al. [15] showed that for ^68^Ga-DOTATOC PET/CT studies *β* values equal to or higher than 400 result in noise levels equal to or lower than those of OSEM (2 min/bp) with improved SNR and SBR. The OSEM reconstruction algorithm used in [15] corresponds to the algorithm recommended by the manufacturer, i.e. GE OSEM. These results are in accordance with our observations when comparing GE OSEM to Q.Clear with 1.5 min/bp. Indeed, Fig. 3a shows that a *β* value around 500 is sufficient to outperform GE OSEM in terms of noise, SNR and SBR (Bonferroni corrected *p* < 0.05). Ter Voert et al. [14] and Svirydenka et al. [22] proposed beta values of 400–550 for ^68^Ga-PSMA, focusing on 2-min emission data of the pelvic region. Longer acquisition times per bed position compared to our study (2–3 min/bp vs 1.5 min/bp in our study) may lead to lower optimal *β* values. In addition, differences in uptake and biodistribution between ^68^Ga-PSMA and ^68^Ga-DOTA tracers and focus on the pelvic region may affect the choice of the *β* factor. Finally, the OSEM reconstruction with 3 iterations, 28 subsets and 5-mm filter used in references [14, 22] could explain the lower *β* values obtained there. Indeed, taking into account that the higher number of subsets used the higher the noise in the resulting image is [23], it would seem that lower *β* values were sufficient to obtain a similar noise level and higher SUV_max_ compared to the OSEM.

Also, the optimal *β* values obtained in our study for ^68^Ga-DOTA examinations are higher than the values obtained by Lindström et al. [19] and Caribé et al. [18] for FDG studies. However, the reference OSEM algorithm used in these studies for comparison with Q.Clear corresponds to the GE OSEM used in present study. Lindström et al. [19] determined an optimal *β* of about 400 using an acquisition time of 3 min/bp. This result is in accordance with our observations when comparing GE OSEM to Q.Clear. Caribé et al. [18] investigated the optimal *β* value to be used for an acquisition time of 1.07 min/bp. As shown above in the “Results” section, our results suggest that Q.Clear with *β* = 700–800 is significantly better than GE OSEM. This concords with Caribé et al.’s [18] results who proposed an optimal value of 750 to outperform OSEM.

There are several limitations to our study. First, the physicians who assessed the images first read a training case in consensus in order to determine the scoring method. For overall image quality, the physicians chose to focus essentially on noise in the images. As a matter of fact, overall image quality and noise level obtained similar scores, regardless of the acquisition time (Fig. 5). This may induce a bias in the scoring, leading to a decrease in final Q.Clear score. Moreover, when rating a new technique versus a well-known one (Hadassah OSEM), observers are more familiar with the latter and consider it as gold standard. Physicians might therefore have been biased and rated Hadassah OSEM outcomes higher than Q.Clear results. Also, the physicians who graded images were blinded to the specific reconstructions and although they were able to compare different reconstructions they did not know the exact lesions. This may have caused a bias in the assessment of tumor detectability. Secondly, only 30 patients were included in this work. This limitation is due to the time consuming task of the reconstruction. Indeed, each study has been reconstructed for different values of *β* and acquisition times for a total of 25 reconstructions per study or 750 reconstructions for the 30 included studies, each reconstruction taking up to 5 min. Therefore, the additional analysis has been performed on a random group of 8 studies. Assessment in a larger patient cohort would allow a more certain implementation in clinical practice. Finally, our study has been conducted on a specific PET/CT system, the Discovery MI. It would be interesting to investigate and confirm the *β* values found here on other systems of the same and different vendors with different PL algorithms.

## Conclusion

^68^Ga-DOTA studies reconstructed with Q.Clear and adequate values of the penalization factor *β* resulted in increased tumor SUV_max_ and in improved SNR and SBR at a similar level of noise compared to our local optimized OSEM (3 iterations, 8 subsets, 6-mm post-processing filter). The optimal *β* value for 1.5 min/bp was 1100–1400 and lead to better image quality than OSEM. Also, Q.Clear allowed to shorten the acquisition time by one-third, resulting in better image quality than OSEM (1.5 min/bp) with *β* = 1300–1600. For 0.5 min/bp, the *β* values assessed did not allow to obtain significantly improved results compared to OSEM. The results of the present study indicate that injected activities or acquisition time can be lowered by one-third for ^68^Ga-DOTA studies when reconstructing the data using the Q.Clear algorithm.

## Data Availability

Patient imaging was done in the scope of the routine clinical diagnostic studies, and the raw data are stored in the hospital archiving system at the Hadassah-Hebrew University Medical Center, Jerusalem, Israel.

## Abbreviations

OSEM: Ordered subset expectation maximization
PL: Penalized-likelihood estimation
bp: Bed position
PET: Positron emission tomography
CT: Computed tomography
TOF: Time-of-flight
GE: General Electric
NEMA: National Electrical Manufacturers Association
IQBP: Image quality body phantom
VPFX-S: VUE Point FX-S algorithm
CR: Contrast recovery
SNR: Signal-to-noise ratio
BV: Background variability
CNR: Contrast-to-noise ratio
SBR: Signal-to-background ratio
